# Efficacy and cost-effectiveness of universal pre-operative iron studies in total hip and knee arthroplasty

**DOI:** 10.1186/s13018-021-02687-w

**Published:** 2021-08-27

**Authors:** Viju Daniel Varghese, David Liu, Donald Ngo, Suzanne Edwards

**Affiliations:** 1 Gold Coast Centre for Bone and Joint Surgery, 14 Sixth Avenue, Palm Beach, Queensland 4221 Australia; 2 Present Address: Department of Orthopaedics, Unit 3, Christian Medical College, Vellore, Tamil Nadu India; 3 Gold Coast University Hospital, 1 Hospital Blvd, Southport, QLD 4215 Australia; 4 Adelaide Health Technology Assessment (AHTA), School of Public Health, University of Adelaide, Adelaide, Australia

**Keywords:** Iron deficiency anaemia, Iron studies, Blood transfusion, Hip and knee replacement

## Abstract

**Background:**

The prevalence of anaemia in patients planned for total hip and knee arthroplasty is about 20%. Optimising pre-operative haemoglobin levels by iron supplementation has been shown to decrease transfusion rates, complications and associated morbidity. The need for universal screening with iron studies of all elective arthroplasty patients is not clearly defined at present.

**Methods:**

Retrospective review of 2 sequential cohorts of patients undergoing primary hip or knee arthroplasty by a single surgeon at a single centre between January 2013 and December 2017. The first group of patients underwent pre-operative iron studies only if found to be anaemic, with a haemoglobin below 12g/dl. From January 2015, all patients irrespective of the presence of anaemia were screened with a complete iron profile before surgery. Patients with a confirmed iron deficiency were administered with intravenous iron prior to surgery. The 2 cohorts were compared with regard to blood transfusion rate post-operatively and cost efficiency for universal screening with iron studies.

**Results:**

There was a net decrease in the allogenic blood transfusion rate from 4.76 to 2.92% when universal iron studies were introduced but the difference was not statistically significant. Obtaining universal pre-operative iron studies is cost neutral with the price of allogenic blood transfusion in a similar cohort. We also diagnosed 5 patients with occult malignancies.

**Conclusions:**

Universal screening with pre-operative iron studies and iron infusion in elective arthroplasty patients may reduce allogenic blood requirements and is cost neutral. An additional benefit is the potential to diagnose asymptomatic malignancies. Further studies are required to show the true benefit of universal pre-operative iron screening.

## Introduction

The prevalence of anaemia among patients undergoing elective total knee (TKA) and hip arthroplasty (THA) has been demonstrated to be significant, ranging between 19 and 44%, and increases with age [1–3]. One of the major risk factors for requiring allogenic blood post-total joint replacement is pre-operative anaemia. Allogenic blood transfusion has been associated with increased post-operative infection rates, length of hospital stay and mortality and morbidity [3]. Pre-operative detection, evaluation and correction of anaemia reduces the need for allogenic blood and the incidence of complications [4]. The fundamental aim of blood management in THA and TKA is to avoid allogenic blood.

Iron deficiency is one of the leading causes of anaemia, accounting for one-third of anaemia cases [5]. However, symptomatic iron deficiency can occur independent of anaemia. Iron deficiency on its own can cause lethargy, may impair pre-operative haemoglobin (Hb) optimisation and delay post-op Hb recovery and is associated with increased post-operative fatigue and hospital stay [6, 7]. In a study of 715 elective orthopaedic patients, 18% of patients were found to be iron deficient in the absence of anaemia [8]. Over half of the body’s iron is stored in Hb, while approximately another 25% is stored intracellularly as ferrous bound to ferritin protein. These two biochemical markers are important indicators for patient recovery in the post-operative period as they reflect the iron levels in the body. The function of ferritin is to replenish iron stores for heme synthesis [9].

Contemporary practice in THA and TKA is to optimise red cell count in the peri-operative period using a variety of methods. These include iron supplementation, erythropoietin and cell salvage re-infusion [10]. Parenteral iron has been shown to be effective by avoiding oral compliance issues and increases Hb levels within 3 weeks. There is however a paucity of evidence available regarding the use of screening iron studies and intravenous iron infusion pre-operatively with its impact on rates of allogenic blood transfusion in elective orthopaedic surgery [11].

We have been utilising a blood conservation strategy algorithm at our institution, which includes pre-operative iron studies in all patients planned for elective THA and TKA surgery from January 2015 [10]. Patients with iron deficiency (serum iron < 5μmol/l and/or serum ferritin < 100 μg/l and TSAT < 20%) were treated with an intravenous iron infusion pre-operatively, irrespective of pre-operative Hb level. The primary aim of this study is to assess whether our protocol of performing universal iron studies on all patients planned for elective joint replacement surgery helped in decreasing transfusion rates. The secondary aim is to assess the cost-effectiveness of pre-operative iron studies in elective THA and TKA.

The study hypothesis is that routine pre-operative iron studies in patients undergoing elective THA and TKA are effective in reducing allogenic transfusion rate and is cost effective.

## Methods

### Study design

A retrospective review of 2 sequential cohorts of patients who underwent primary THA or TKA under a single surgeon between January 2013 and December 2017 was performed. Universal screening for iron deficiency of all patients irrespective of Hb level was introduced as part of the pre-operative protocol from January 2015. Patients were considered to be iron deficient if they had a serum iron < 5μmol/l and/or serum ferritin < 100 μg/l and TSAT <20% and were treated with an intravenous iron infusion pre-operatively, irrespective of pre-operative Hb level. Prior to this, only patients found to be anaemic, defined as Hb level less than 12g/dl, were investigated with iron studies and this formed the comparator control group for the study. Between the 2 sequential groups of patients, no other change in patient management or surgical technique was introduced and the surgical time was comparable. Data was collected by review of the hospital records and surgeon’s electronic patient files. Ethics approval for the study was obtained by the hospital Research and Ethics Committee.

Demographics of the two study populations are shown in Table 1. The iron studies comprised serum iron, serum ferritin, serum transferrin, serum total iron binding capacity (TIBC) and serum transferrin saturation (TSAT). Patients with studies indicating iron deficiency were referred to a haematologist, who investigated the cause and administered intravenous iron infusion as appropriate pre-surgery. Iron transfusions comprised a single dose of 500–1000mg intravenous ferritin carboxy maltose, administered in a day stay unit, given a minimum 4 weeks before surgery. The primary outcome measure of the study is the blood transfusion rate between the 2 cohorts. Secondary outcome measures such as pre- and post-operative Hb levels, per-operative blood loss and rates of iron transfusion were also collected. Data in the universal iron screening cohort, regarding patients who were diagnosed to have incidental malignancies on further investigation, was also collected.

**Table 1:** Comparison of cohorts pre- and post-universal screening

	2013–2015 (pre-universal screening group)	2015–2017 (universal screening group)	*P*-value
Number	420	514	
Age, mean (SD)	71.1 (9.26)	71.7 (9.51)	0.3324
Gender (M:F)	200:220	239:275	0.9805
Side (right:left)	195:225	271:243	0.0484
THA:TKA	220:200	296:218	0.9151
BMI, mean (SD)	30.12 (5.60)	29.56 (5.67)	0.1317
Haemoglobin (pre-op), mean (SD)	134.97gm/dl (12.33)	138.33gm/dl (12.91)	0.0001
Iron deficiency anaemia	105 (25%)	180 (35%)	0.7829
Iron infusion rate	21 (5%)	71 (13.8%)	<0.0001
Blood transfusion rate	20 (4.8%)	15 (2.9%)	0.0886

All THA were performed through an anterolateral approach in the lateral position. Uncemented acetabular and femoral components were used, with no drain. All TKA were performed using computer navigation without a tourniquet, using cemented components and routine patella resurfacing. A subcutaneous suction drains outside the joint cavity was used in TKA, with the drain removed within 24 h after surgery. Intra-operative cell salvage was used in all cases with autologous re-infusion if sufficient blood was salvaged. After implantation of components, topical tranexamic acid (3g diluted in 20 ml N/saline) was instilled for 5 min to the surgical site prior to final lavage and closure, followed by 1 g orally at 3 and 6 h post-operatively.

Patients were ambulated on the day of surgery. Venous thromboembolism prophylaxis comprised of enoxaparin 40mg daily commenced 4 h post-operatively until discharge from hospital. Patients were discharged on aspirin for 6 weeks post-operatively. For patients on warfarin pre-operatively, this was continued during the peri-operative period. Warfarin dose was adjusted leading to surgery, aiming for an international normalisation ratio (INR) of 2 on the day of surgery. The warfarin was restarted the night of surgery, with bridging enoxaparin if the INR fell below 2.

Data collected included demographics, body mass index (BMI), pre-operative haemoglobin (early Hb), iron parameters (serum iron, total iron binding capacity, serum transferrin and serum ferritin), haemoglobin post-iron transfusion (late Hb) and post-operatively (post-op Hb), peri-operative blood loss, type of anaesthesia, ASA grade and type of anticoagulation, total blood loss, blood collected by cell salvage and Hb change between pre-operative and day 2 post-operative. Anaemia in our study is defined as Hb less than 13g/dl in males and less than 12g/dl in females. The transfusion trigger post-operatively was Hb less than 8g/dl or symptomatic anaemia with Hb less than 10g/dl and co-existing comorbidities

### Statistical methods

Descriptive statistics for iron variables were presented for all data and by iron transfusion (performed or not performed), depending on the distribution of the data with mean and standard deviation presented for normally distributed variables. Independent *t*-tests were performed to compare continuous iron variables between the two cohorts.

Bivariate binary logistic regressions were performed for the outcome: blood transfusion versus demographic and blood-related predictors. A multivariable model was performed including previous iron transfusion and variables with *P* value < 0.2 on bivariate regression.

Bivariate linear regressions were performed for the outcomes: blood loss, RBC collected and HB drop (in separate models) versus demographic and blood-related predictors. A multivariable model was performed including previous iron transfusion and variables with *P* value < 0.2 on bivariate regression. Assumptions of a linear model were checked by inspection of histograms and scatter plots of residuals and predicted values.

A cross tabulation was performed for iron deficiency anaemia versus general anaemia, with an associated chi-square *P* value.

## Results

Nine hundred and thirty-four patients from 2013 to 2017 were included in the study. There were 420 patients in the group prior to routine iron studies (control group) and 514 in the cohort who underwent routine iron deficiency screening (treatment group). The overall blood transfusion rate in the control group was 20/420 (4.8%), and in the treatment group, it was 15/514 (2.9%) with 5 following THA and 10 following TKA. Table 1 shows the demographics, iron deficiency anaemia rates, iron transfusion and blood transfusion rates between the 2 cohorts. There were no major/minor side effects following the iron transfusion. There was an increase in the average Hb levels of 0.34g/dl because of the universal screening protocol which was statistically significant (*P* < 0.0001). Though there was a reduction in blood transfusion rates, this was not statistically significant (chi-square *P* value = 0.0980).

In the 514 patients in the treatment group, 9.2% were detected to have anaemia of which 30% were iron deficient. Among the treatment cohort with normal Hb, 34.6% were found to be iron deficient (Table 2). Iron infusions were administered to 71 of 514 (13.8%) patients, with 14.4% in THA and 13.5% in TKA.

**Table 2:** Prevalence of anaemia and iron deficiency in joint replacement patients

	Iron deficiency	Iron deficiency	Total	*P* value
Anaemia	Yes	No		0.5990
Yes	12	27	39 (9.24%)	
No	134	249	383 (90.76%)	
Total	146 (35%)	276 (65%)	422	

Tables 3 and 5 outline the distribution of iron indices, early, late and post-operative haemoglobin rates among the hip and knee replacement groups, respectively, and in those patients, who had iron infusion as opposed to those who did not. These results show that there was a significant improvement in the Hb levels of around 0.3g/dl following the infusion protocol (Tables 4 and 5). The decrease in the Hb levels post-operatively was similar in the iron transfusion group and non-iron transfusion group. Tables 4 and 6 show comparable demographics for age, BMI and blood loss for both hip and knee cohorts.

**Table 3:** Descriptive statistics for iron variables comparing the iron transfusion group versus the non-iron transfusion group. *TKA* total knee arthroplasty

Iron variable	All data (mean (SD)) *N* = 296	Iron transfusion (mean (SD)) *N* = 38	No iron infusion (mean (SD)) *N* = 258	Independent *T*-test *P* value
S iron (5–30 μmol/l)	15.5 (5.1)	11.9 (4.1)	16.1 (5.0)	<0.0001
Transferrin (1.9–3.1g/l)	2.5 (0.4)	2.8 (0.4)	2.5 (0.3)	0.0013
TIBC (47–77 μmol/l)	62.8 (8.9)	69.0 (10.8)	61.9 (8.2)	0.0015
Tran satrn (20–45%)	25.7 (10.7)	18.1 (6.4)	26.9 (10.7)	<0.0001
Ferritin (30–300 μg/l)	176.1 (170.3)	51.0 (30.4)	196.0 (175.0)	<0.0001
Early Hb (g/dl)	138.81 (11.63)	130.29 (12.22)	140.18 (10.95)	<0.0001
Late Hb (g/dl)	138.35 (12.04)	132.25 (13.01)	139.22 (11.67)	0.0011
Post-op Hb (g/dl)	112.18 (13.38)	105.41 (14.44)	113.22 (12.93)	0.0009
Intra-operative blood salvage rate	47.6%	45%	52%	0.4211*

*Chi-square test *P* value

**Table 4:** TKA data: descriptive statistics of demographics

Variable	Mean	Standard deviation
Current age	72.40	8.47
BMI	30.20	5.78
Blood loss	331.51	144.63
RBC collected	176.82	98.54
Hb drop	2.65	0.84

**Table 5:** Descriptive statistics for iron variables comparing the iron transfusion group versus the non-iron transfusion group. *THA* total hip arthroplasty

Iron variable	All data (mean (SD)) *N* = 216	Iron transfusion (mean (SD)) *N* = 33	No iron infusion (mean (SD)) *N* = 183	Independent *T*-test *P* value
S iron (5–30 μmol/l)	15.4 (5.2)	13.1 (4.7)	15.9 (5.1)	0.0067
Transferrin (1.9–3.1g/l)	2.5 (0.4)	2.7 (0.3)	2.4 (0.30)	<0.0001
TIBC (47–77 μmol/l)	62.0 (8.9)	68.3 (8.0)	60.7 (8.5)	<0.0001
Tran satrn (20–45%)	25.6 (9.3)	19.8 (8.6)	26.8 (9.0)	0.0001
Ferritin (30–300 μg/l)	181.5 (149.2)	59.8 (54.4)	208.5 (150.2)	<0.0001
Early Hb (g/dl)	138.95 (13.64)	130.39 (14.05)	140.64 (12.95)	0.0002
Late Hb (g/dl)	138.30 (14.05)	134.12 (13.23)	139.08 (14.10)	0.0626
Post-op Hb (g/dl)	117.26 (13.44)	111.55 (11.33)	118.26 (13.56)	0.0100
Intra-operative blood salvage rate	53.2%	60%	50%	0.2912*

*Chi-square test *P* value

**Table 6:** THA data: descriptive statistics of demographics

Variable	Mean	Standard deviation
Current age	68.48	9.91
BMI	28.67	5.40
Blood loss	324.78	133.32
RBC collected	168.72	81.91
Hb drop	2.07	0.85

Iron infusions were found to be predictive for blood transfusions among TKR patients but not among THR patients (Tables 7 and 8). Low pre-operative Hb was also strongly predictive of blood transfusion rates. Other factors that were positively associated with increased peri-operative blood loss and lower post-op Hb included general anaesthesia, use of warfarin and rivaroxaban as anticoagulants and female gender (Tables 7 and 8).

**Table 7:** Factors affecting blood transfusion/blood loss in TKA

Total knee arthroplasty data	OR/estimate (95% CI)	Comparison *P* value	Global *P* value
Bivariate analysis^a^			
Previous iron transfusion	13 (3, 56)		0.0007
Multivariable analysis for outcome: blood transfusion^a^			
Late pre-operative haemoglobin	0.80 (0.70, 0.91)^a^		0.0010
Multivariable analysis for outcome: blood loss			
Type of anaesthesia			0.0111
Spinal/GA vs GA	−111.4 (−185.6, −37.4)	0.0032	
Spinal vs GA	−71.3 (−135.2, −7.5)	0.0286	
Anticoagulants			<.0001
0 vs 2^b^	−274 (−417, −130)	0.0002	
0 vs 3^b^	−232.7 (−403.0, −62.5)	0.0074	
Multivariable analysis for outcome: haemoglobin drop			
BMI	−0.02 (−0.04, 0.0)		0.0120
Gender (male vs female)	−0.35 (−0.56, −0.15)		0.0007
Anticoagulants			0.0288
0 vs 2^b^	−0.67 (−1.26, −0.08)	0.0249	
1 vs 2^b^	−1.44 (−2.41, −0.48)	0.0034	
Late pre-operative haemoglobin	0.02 (0.01, 0.03)		<.0001

^a^Binary logistic regression – odds ratio (95% confidence interval); modelling the probability that blood transfusion = ‘Yes’

^b^Anti coag (0 = Clexane, 1 = aspirin, 2 = warfarin, 3 = all others [rivaroxaban, apixaban])

**Table 8:** Factors affecting blood transfusion/blood loss in THA

Total hip arthroplasty data	OR/estimate (95% CI)	Comparison *P* value	Global *P* value
Multivariable analysis for outcome: blood transfusion^a^			
Late pre-operative haemoglobin	0.89 (0.83,0.95)		0.0006
Multivariable analysis for outcome: RBC collected			
Iron transfusion, yes vs no	60.2 (22.3, 98.1)		0.0019
Type of anaesthesia			
Spinal vs GA	−38.7 (−68.9, −8.4)		0.0375
Late pre-operative haemoglobin	1.46 (0.43, 2.50)		0.0056
Multivariable analysis for outcome: blood loss			
Iron transfusion, yes vs no	85.3 (11.7, 158.8)		0.0231
Anticoagulants			0.0496
0 vs 3^b^	-162.6 (−294.1, −31.1)	0.0153	
2 vs 3^b^	−182.4 (−363.1, −1.7)	0.0478	
Multivariable analysis for outcome: Hb drop			
Late pre-operative haemoglobin	0.02 (0.01,0.03)		<0.0001

^a^Binary logistic model – odds ratio (95% CI) – modelling the probability that blood transfusion = yes

^b^Anti coag (0 = Clexane, 1 = aspirin, 2 = warfarin, 3 = all others [rivaroxaban, apixaban])

Among our elective THA and TKA cohorts, we diagnosed 10 patients, following further evaluation of their iron deficiency with underlying gastric causes or malignant disease which were subsequently treated prior to joint arthroplasty surgery. These are listed in Table 9.

**Table 9:** Pathology diagnosed following evaluation of iron deficiency anaemia

Gastric erosions/gastric ulcer	5
Bowel cancer	3
Haematological cancer	2 (1 myelodysplastic syndrome, 1 chronic myeloid leukaemia)

For cost-effectiveness of routine pre-operative iron studies, we calculated the numbers needed to treat (NNT) of our cohort as 49.06. This is derived from the formula, NNT = 1/incidence in the control group − incidence in the treated group.

For iron infusion: NNT = 71/514 × 49.06 = 6.8

Cost for iron infusion: 6.8 × $135 (per iron infusion) = $ 918 ($ = Australian dollar/AUD)

For iron studies: NNT = 433/514 × 49.06 = 41.33

Cost for iron studies: 41.33 × $30 (per iron study) = $ 1240.

Total costs for iron protocol = iron infusion costs + iron studies costs ($918 + $1240) = $2158

For blood transfusion: (2 × 15/512) × 49.06 = 2.86 units (each patient had 2 units of blood)

Costs for blood transfusion: 2.86 × $700 (per blood unit) = $2004

Hence, our universal iron screening and iron infusion protocol are cost neutral with the expense of allogenic blood transfusion ($2158 vs $2004) in this patient cohort.

## Discussion

Universal screening with pre-operative iron studies and iron infusion in elective THA and TKA patients in our study decreased allogenic transfusion rates post-operatively but was not statistically significant. We believe part of the reason why the allogenic transfusion rate was not significant between the 2 groups is our transfusion rate was already very low in the control patients, because of our blood management protocols [12]. Universal screening with iron studies was essentially equivalent cost compared to the savings from reduction in blood transfusion rates. However, we believe pre-operative iron screening is still advantageous, as our cost calculation did not factor into account possible complications and increased length of hospital stay which often result from allogenic blood transfusion.

Pre-operative anaemia occurred in 1 out of 10 patients presenting for THA and TKA, of which iron deficiency anaemia was the cause in one-third, which is similar to the results of other studies [13]. Intravenous iron infusion rapidly increases the pre-operative Hb levels and bypasses the effects of oral compliance and the effect of hepcidin in patients with anaemia of chronic disease. It can help to decrease allogenic transfusion rates, reduce post-op Hb drop after surgical blood loss and hasten the post-operative recovery rate of Hb. The average decrease in Hb following THA and TKA is approximately 4g [14]. In our treatment group, we were able to increase the Hb pre-operatively by average 0.2 g in TKA and 0.3g in the THA cohort. Moreover, the universal screening protocol (2015–2017) significantly increased the average Hb by a mean of 0. 3g/dl (*P* < 0.0001), as compared to the cohort prior to 2015.

One-third of our patients who had normal Hb had iron deficiency, and this is similar to recent studies [15]. There are 2 important ramifications of this finding. Firstly, iron deficiency on its own, even in the absence of anaemia, can cause lethargy and tiredness. This may then hinder a patient’s ability to rehabilitate following surgery [6]. Secondly, iron deficiency is a sign of potential malignancy, especially after the age of 60. Eight to 15% of patients with iron deficiency anaemia will have a gastrointestinal (GI) malignancy [16], and colorectal carcinoma presents with iron deficiency anaemia in 50% of cases [17]. More importantly, patients with iron deficiency alone in the absence of anaemia have been found to have an increased risk of GI malignancy after 2 years [18]. Following the commencement of routine iron screening in our elective THA and TKA patients, we diagnosed 5 malignancies in patients who were otherwise asymptomatic and unaware of their diagnosis. The incidental benefit of diagnosing malignancies early in these patients cannot be discounted, which would not have been possible without universal iron studies which helped us identify these patients and instigate early treatment.

As has been demonstrated in previous studies, gender (female), type of anaesthesia (GA), type of surgery (THR) and low pre-operative Hb were associated with increased allogenic blood transfusion risk [19, 20]. Interestingly, we were able to show that pre-operative iron deficiency correlated with blood loss and iron transfusions were a strong prognostic factor for having post-op blood transfusions. This was seen only on bivariate analysis and in the TKA group. The higher blood transfusion rates among patients who underwent iron transfusion could probably be explained by the lower baseline Hb levels and impaired erythropoiesis. Of the 14 patients transfused, 3 had blood disorders and 4 had multiple comorbidities with anaemia of chronic disease and consequent impaired erythropoiesis, while 4 had ischemic heart disease with stents with a low transfusion trigger.

The weaknesses of this study include the retrospective design and method of data collection via medical record review. There may have been recall bias and inaccurate data entry into the medical record which could have affected the results. Our findings may not be applicable to all regions of the world or all patient ethnicities. Additionally, as the study is a single surgeon series, surgical techniques and protocols may also vary and influence the outcomes and conclusions. Finally, as our allogenic transfusion rate in the control group was already low, the study may be underpowered and hence we were unable to demonstrate statistical significance with allogenic blood transfusion rates. Nevertheless, we believe our findings still highlight several important issues and are relevant to surgeons undertaking THA and TKA, especially so at present, where the focus has shifted to short stay or day care surgery. Correcting pre-operative iron deficiency anaemia, through universal iron studies, may facilitate this. The study is a single surgeon series with a uniform protocol and no change in surgical pathway apart from iron studies between the 2 comparison groups.

Transfusion rates for joint replacements have been reported to be about 18% for knee and 22% for hip arthroplasties [21], but can range from 13 to 87%, showing [22] the high variability in clinical practice. Our low allogenic transfusion rates were decreased even further by routine pre-operative iron studies and iron infusion protocol. This could also partly explain why we were not able to show a statistically significant decrease in blood transfusion rates or cost-effectiveness. While Medicare cost of blood transfusion is $700 per unit, the cost of maintaining a blood bank and personnel make the actual costs of a unit of blood about 2–5 times more [23]. The true cost of allogenic blood should also factor complications and increased length of hospital stay often associated. Adding hidden costs and having a larger study group might have shown cost-effectiveness with our protocol. A recent systematic review investigating pre-operative iron transfusions showed a definite decrease in blood transfusions, length of hospital stay and post-operative infections [24].

## Conclusion

Universal screening with pre-operative iron studies and iron infusion in elective total hip and knee arthroplasty patients may reduce allogenic blood requirements and is cost neutral. An additional benefit is the potential to diagnose asymptomatic malignancies. Further prospective studies with greater power may be required to show the true benefit of universal pre-operative iron screening by factoring into account length of hospital stay, cost of complications and maintenance of the blood bank, and cost benefits of early diagnosis of occult malignancies.

## Data Availability

The datasets used and/or analysed during the current study are available from the corresponding author on request.

## Abbreviations

TKA: Total knee arthroplasty
THA: Total hip arthroplasty
Hb: Haemoglobin
TIBC: Total iron binding capacity
TSAT: Transferrin saturation
BMI: Body mass index
ASA: American Society of Anesthesiologists
GA: General anaesthesia
NNT: Numbers needed to treat
GI: Gastrointestinal
